# Ginsenoside Rb1 Improves Atherosclerosis by Inhibiting Endothelial Cell Pyroptosis

**DOI:** 10.1155/omcl/6137635

**Published:** 2026-04-29

**Authors:** Xuejiao Jiang, Tianqi Jiang, Zhaoyu Pan, Chun Chen, Xinyu Ji, Xiongyao Wang, Wenjing Zong

**Affiliations:** ^1^ College of Traditional Chinese Medicine, Inner Mongolia Medical University, Hohhot, China, immu.edu.cn; ^2^ Department of Orthopedics, Inner Mongolia People’s Hospital, Hohhot, China, nmgyy.cn; ^3^ Department of Otolaryngology Head and Neck Surgery, Xiangya Hospital, Central South University, Hunan, China, csu.edu.cn; ^4^ Institute of Basic Research in Clinical Medicine, China Academy of Chinese Medical Sciences, Beijing, China, cacms.ac.cn; ^5^ Institute of Basic Theory for Chinese Medicine, China Academy of Chinese Medical Sciences, Beijing, China, cacms.ac.cn

**Keywords:** atherosclerosis, caspase-1, endothelial cell, ginsenoside Rb1, pyroptosis

## Abstract

**Purpose:**

This study aimed to investigate the effect of ginsenoside Rb1 (Gs‐Rb1) on endothelial cell (EC) pyroptosis in atherosclerosis (AS).

**Method:**

ApoE^−/−^ mice and mouse aortic endothelial cells (MAECs) were used as research subjects. An in vivo AS model was established by feeding ApoE^−/−^ mice a high‐fat diet (HFD) for 3 months, followed by intragastric administration of Gs‐Rb1 at 40 mg/kg/day for 3 months. Pathological changes were evaluated by hematoxylin‐eosin (HE) and Oil Red O staining. Caspase‐1 expression was detected by immunofluorescence. Pyroptosis‐related protein and mRNA levels were measured by Western blotting and RT‐PCR. Inflammatory factors (IL‐18 and IL‐1β) and LDH were quantified by ELISA. For in vitro experiments, MAECs were stimulated with oxidized low‐density lipoprotein (ox‐LDL, 150 μg/mL) to induce pyroptosis, followed by treatment with Gs‐Rb1 (60 or 80 μg/mL) for 12 h. Cell death was assessed by flow cytometry.

**Result:**

Gs‐Rb1 significantly reduced aortic plaque area in mice. It decreased the expression of pyroptosis‐related proteins (caspase‐1, cleaved caspase‐1, GSDMD, and NLRP3) and mRNA levels in aortic tissues. Serum levels of LDH, IL‐18, and IL‐1β were also significantly reduced. In vitro, Gs‐Rb1 reduced cell death rate and inhibited pyroptosis in endothelial cells (ECs).

**Conclusion:**

This study confirms the therapeutic effect of Gs‐Rb1 on AS at both animal and cellular levels. Inhibition of EC pyroptosis may be the key mechanism underlying the anti‐atherosclerotic effects of Gs‐Rb1.

**Summary**

This study provides new evidence that ginsenoside Rb1 attenuates atherosclerosis by reducing EC pyroptosis.

## 1. Introduction

Atherosclerosis (AS) is the main pathological basis of various vascular diseases such as coronary heart disease and stroke [1]. AS is characterized by lipid deposition, plaque formation, and narrowing of the arterial space, which can lead to ischemia or necrosis of tissues and organs [2, 3]. Current studies indicate that the major cells involved in AS include endothelial cell (EC), vascular smooth muscle cells, macrophages, and lymphocytes. ECs form a selectively permeable monolayer that regulates paracellular and transcellular transport, and actively participates in paracrine signaling, vascular tone regulation and immune modulation; professional phagocytosis is primarily performed by macrophages [4]. EC injury and dysfunction are key factors in the formation of AS [5].

Pyroptosis is a type of programed cell death in which cells continue to swell until their membranes rupture, causing release of intracellular contents and triggering a robust inflammatory response [6]. The key pathway for pyroptosis is NLRP3 (nucleotide‐binding domain, leucine‐rich‐containing family, pyrin domain‐containing‐3) inflammasome activates caspase‐1, which cleaves gasdermin D (GSDMD) and causes perforation of the cell membrane [7]. A large number of studies have shown that EC pyroptosis plays a key role in the development of AS [8].

As a representative of traditional Chinese medicine, ginseng (*Panax ginseng* C. A. Meyer) is derived from the root of the Araliaceae plant ginseng. Ginseng is a Chinese herbal medicine widely used around the world [9] and its main function is to tonify “qi” and nourish “yin”. Studies have found that ginseng contains a variety of active ingredients. Ginsenoside Rb1 (Gs‐Rb1) is the main pharmacologically active component of ginseng, accounting for more than 20% of total ginsenosides [10]. It has previously been observed that Gs‐Rb1 can resist oxidation, inhibit inflammation, regulate autophagy, and reduce apoptosis [11]. It has been recently demonstrated that Gs‐Rb1 has significant potential in the treatment of cardiovascular diseases [12, 13]. A recently registered systematic review protocol [14] aims to evaluate the effects of ginseng on cardiovascular disease risk factors, including inflammatory and oxidative stress markers. This ongoing review highlights the growing interest in clarifying the molecular mechanisms underlying ginseng’s cardioprotective effects. Our study, by investigating the role of Gs‐Rb1 in inhibiting endothelial pyroptosis—a newly recognized inflammatory form of programed cell death—provides mechanistic evidence that may complement and extend the findings of this systematic review. Specifically, our findings on IL‐1β, IL‐18, and NLRP3 inflammasome regulation could contribute to the inflammatory pathway analysis within the review framework. However, it is still unknown whether Gs‐Rb1 can inhibit EC pyroptosis.

Although the anti‐inflammatory properties of Gs‐Rb1 have been widely reported, its potential interaction with inflammasome pathways remains underexplored. A few recent studies have suggested that Gs‐Rb1 may modulate inflammasome activation. For example, Gs‐Rb1 was shown to suppress NLRP3 inflammasome activation in LPS‐induced macrophages, leading to reduced IL‐1β secretion [15]. Additionally, Gs‐Rb1 has been found to inhibit ROS generation and NF‐κB signaling, both of which are upstream regulators of the NLRP3 inflammasome [16]. These findings imply that Gs‐Rb1 may exert its anti‐inflammatory effects, at least in part, by interfering with inflammasome‐mediated pyroptosis. However, whether Gs‐Rb1 can directly inhibit endothelial pyroptosis via the NLRP3/caspase‐1/GSDMD axis in the context of AS has not been investigated. Therefore, this study aims to explore the potential role of Gs‐Rb1 in modulating endothelial pyroptosis and its underlying mechanisms in AS.

Based on the above background, we speculate that Gs‐Rb1 can inhibit AS by reducing pyroptosis levels and verified this assumption through in vitro and in vivo experiments.

## 2. Experimental Materials and Methods

### 2.1. Animal Ethics

The study was reviewed by the Ethics Committee of Inner Mongolia Medical University on 15 March 2024. (YKD20240217). Animals were housed five per cage and maintained at a constant temperature (25 ± 3°C) and humidity (60 ± 10%), with a 12 h light/dark cycle, and provided with free access to food and water.

### 2.2. Materials

Gs‐Rb1 (Cat: B21050, purity ≥98%, Lot: 20231201; Shanghai Yuanye Bio‐Technology, China). IL‐1β antibody (AB clonal, A1112, 1:1000); IL‐18 antibody (AB clonal, A 1115, 1:1000); GSDMD antibody (AB clonal, A 18281, 1:1000); Caspase‐1 antibody (CST, 83383, 1:1000); Cleaved caspase 1 antibody (CST, 89332, 1:1000); NLRP3 antibody (CST, 15101, 1:1000); CD31 (Santa Cruz, sc‐1506, 1:100); the IL‐1β (#EK0394); and IL‐18 (#EK0365) ELISA Kits were purchased from Boster Ltd.

### 2.3. Animals

The mice (male, 6‐week‐old, 18–22 g) were obtained from Beijing Vitong Lihua Experimental Animal Technology Co. Ltd. (Beijing, China). The ApoE^−/−^ mice were randomly assigned to three groups after 1 week of adaptive feeding: normal control group (NC, C57BL/6J, 5 mice per group, standard diet), model control group (MC, ApoE^−/−^, 5 mice per group, high‐fat diet (HFD)), and Gs‐Rb1 group (ApoE^−/−^, 5 mice per group, and HFD). After 3 months of feeding, different treatments were given. The Rb1 group was administered Rb1 at 40 mg/kg/d. The dose of Gs‐Rb1 (40 mg/kg/day) was selected based on previous preclinical studies demonstrating significant anti‐atherosclerotic and anti‐inflammatory effects at this level in ApoE^−/−^ mice [17, 18]. The remaining two groups were gavaged with the same volume of normal saline. The treatment lasted 3 months.

### 2.4. Cell Processing

Mouse aortic endothelial cells (MAEC) were cultured under standard cell conditions: 37°C, 5% CO_2_, the culture medium containing Dulbecco’s modified Eagle’s medium (DMEM) was purchased from Life Technologies (Thermo Fisher Scientific), supplemented with 10% fetal bovine serum (FBS) was purchased from Gibco (Mulgrave, Australia), and 1% penicillin/streptomycin. MAECs were used between passages 3 and 5 to ensure phenotypic stability and consistent response to pyroptosis stimuli. MAECs (passages 3–5) were seeded at 1 × 10^4^ cells/well in 96‐well plates. After 12 h, medium was replaced with fresh DMEM containing ox‐LDL (50, 100, 150, or 200 μg/mL) or vehicle. Following 12 h stimulation, cell viability was assessed using CCK‐8 (30 min, 37°C) according to the manufacturer’s instructions (Vazyme, Nanjing, China; Cat: CK8‐02).

### 2.5. Pathological Observation

Mouse aorta tissue was collected and cryosectioned. We performed Hematoxylin/eosin (HE) and Oil Red O staining for lesion analysis. We used light microscopy to observe the tissue structure and quantitatively analyzed the patch area.

### 2.6. Enzyme‐Linked Immunosorbent Assay

We used enzyme‐linked immunosorbent assay (ELISA) kits to detect the release levels of lactate dehydrogenase (LDH). In brief, mouse serum and cell supernatant were collected and performed detection according to the instructions of the kit.

### 2.7. Immunofluorescence Staining

We detected the fluorescence expression of caspase‐1 and CD31 in mouse aorta tissue. Use the 5% bovine serum albumin solution to block the tissue and incubate with the corresponding primary antibody. Then washed the slice with PBS (3 times) and incubated them with corresponding secondary antibodies at room temperature. We washed the slice and observed with a fluorescence microscope.

### 2.8. Cell Death Detection

Cells were digested using 0.25% trypsine‐EDTA and collected by centrifugation (1000 rpm, 5 min). Wash cells twice with cold PBS and then resuspend cells in the 1 × binding buffer. Add 5 μL FITC Annexin and 5 μL PI. The cells were gently vortexed and incubated for 15 min at room temperature in dark. Analyze by flow cytometry in 1 h.

### 2.9. Real‐Time PCR

Use Trizol reagent (NCM Biotech, Suzhou, China) to extract total RNA from tissues and cells. The total RNA was then reversed into cDNA using the kit (Vazyme; Beijing, China; Cat: R323). Quantification was performed using real‐time PCR with SYBR Green Master Mix (Vazyme; Beijing, China; Cat: Q712‐02). Then the expression levels of pyroptosis genes were analyzed. Primer sequences are shown in Supporting Information 1: (Table S1).

### 2.10. Western Blotting Analysis

Total protein was extracted from tissues and cells, and the concentration was adjusted. The proteins were separated using sodium dodecyl sulfate polyacrylamide gel electrophoresis (SDS‐PAGE) and transferred to polyvinylidene fluoride membranes (Millipore, IPVH00010). Incubate with the corresponding primary and secondary antibodies after 2 h of blocking. Then observe and compare protein expression.

### 2.11. Data Analysis

Data are presented as mean ± SEM. *n* = 5 biological replicates per group. Statistical differences were analyzed by one‐way ANOVA followed by Tukey’s post‐hoc test using GraphPad Prism 9.0 (GraphPad Software, San Diego, CA, USA).  ^∗^ *p* < 0.05 was considered significant.

## 3. Results

### 3.1. General Observations

During the 12‐week treatment period, no drug‐related mortality or overt adverse reactions (diarrhea, ruffled fur, behavioral abnormalities) were observed. Body weight increased gradually in all groups, with no significant difference between the MC and the Gs‐Rb1 groups at any time point (Supporting Information 2: Figure S1A). Gs‐Rb1 was not administered to NC (C57BL/6J) mice because the aim was to evaluate therapeutic—rather than preventive—effects under hyperlipidaemic conditions.

### 3.2. Ginsenoside Rb1 Can Reduce Plaque Area in ApoE^−/−^ Mice

To observe the effect of Gs‐Rb1 on the area of aortic plaque in mice, we performed HE and Oil Red O staining on the aortic root of mice. The experimental results showed that a HFD can significantly induce the formation of aortic root plaque and increase the plaque area in ApoE^−/−^mice. The lipid deposition in the plaques of mice in the MC group was obvious. The plaque area decreased after Gs‐Rb1 treatment (Figure 1).

Figure 1Gs‐Rb1 can reduce the plaque area in the aortic root of mice. (a, b). HE and Oil Red O pathological staining. (c) Data analysis of plaque area. Scale bar = 250 μm, Magnification: ×50. Scale bar = 100 μm, Magnification: ×100. NC, normal control group; MC, model control group; Gs‐Rb1, ginsenoside Rb1 group.  ^∗^ *p* < 0.05,  ^∗∗∗^ *p* < 0.001, *n* = 3 biologically independent animals per group for all datasets shown in this panel (NC, MC, and Gs‐Rb1).

**Figure:**
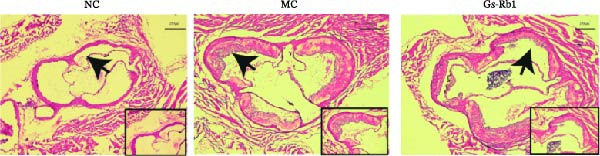
(a)

**Figure:**
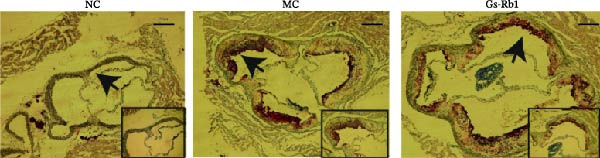
(b)

**Figure:**
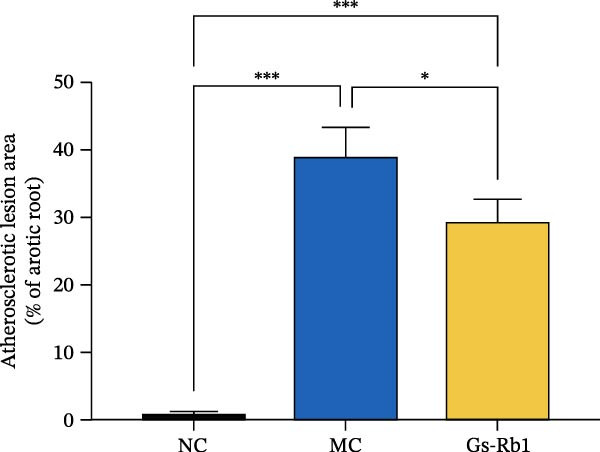
(c)

### 3.3. Ginsenoside Rb1 Reduced Pyroptosis Features of Endothelial Cells in Mice Aorta

**Figure 2:**
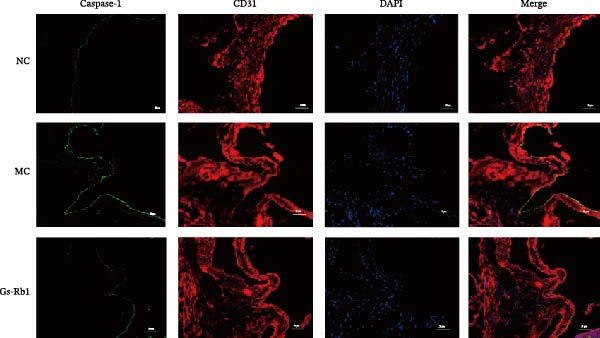
Fluorescence intensity expression of caspase‐1 (green) and CD31 (red) in mouse aorta. Scale bar = 100 μm, Magnification: ×100.

Activation of caspase‐1 is the key to pyroptosis [6]. Observation of EC pyroptosis in mouse aorta by dual staining of caspase‐1 and CD31. The experimental results showed that caspase‐1 was significantly activated in the MC group. The number of cell deaths in mice induced by HFD was significantly increased. However, Gs‐Rb1 treatment can significantly reverse these changes (Figure 2).

### 3.4. Ginsenoside Rb1 Reduced the Expression of Key Proteins and Genes in the Classical Pyroptosis Pathway

To observe the expression of pyroptosis in ApoE^−/−^ mice, we detected the expression levels of pyroptosis‐related proteins and genes in aortic tissue by Western blot and Real‐time PCR. The experimental results showed that the expressions of pyroptosis‐related proteins were significantly increased in the MC group. Furthermore, after treatment by Gs‐Rb1, the expression of pyroptosis decreased in different degrees. For example, in the Gs‐Rb1 group, caspase‐1 protein expression was only 76% of MC; IL‐1β was 48.8%; IL‐18 was 55.8%; NLRP3 was 85.7%; cleaved caspase‐1 was 61.8%; pro‐GSDMD was 59.6%; and cleaved GSDMD was 53.4%. Meanwhile, the gene expression of pyroptosis had consistent changes (Figure 3a–h). In the Gs‐Rb1 group, gene expression of caspase‐1 was only 43.8% of MC; IL‐1β was 45.5%; IL‐18 was 55.9%; NLRP3 was 90.5%; and GSDMD was 59.4% (Figure 3i–m).

Figure 3Gs‐Rb1 can reduce pyroptosis levels in AS. (a–h) Protein levels of pyroptosis‐related proteins in ApoE^−/−^ mice. (i–m) Gene expression levels of pyroptosis‐related genes in ApoE^−/−^ mice. (n, o) The levels of IL‐1β and IL‐18 in ApoE^−/−^ mice serum. (p) Effects of Gs‐Rb1 on LDH release in mice. ^∗^ *p* < 0.05,  ^∗∗^ *p* < 0.01,  ^∗∗∗^ *p* < 0.001, NS, non‐significant, *n* = 5 biologically independent animals per group for all datasets shown in this panel (NC, MC, and Gs‐Rb1).

**Figure:**
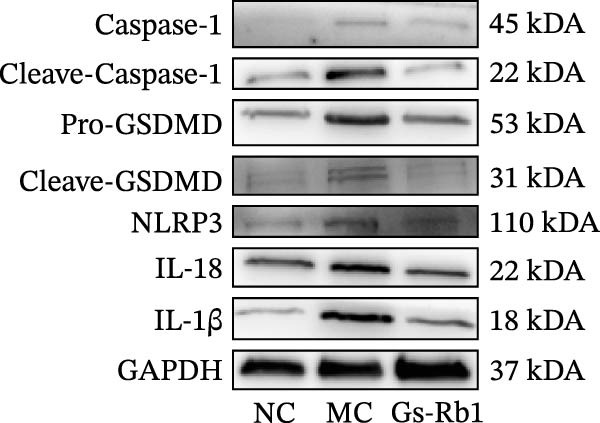
(a)

**Figure:**
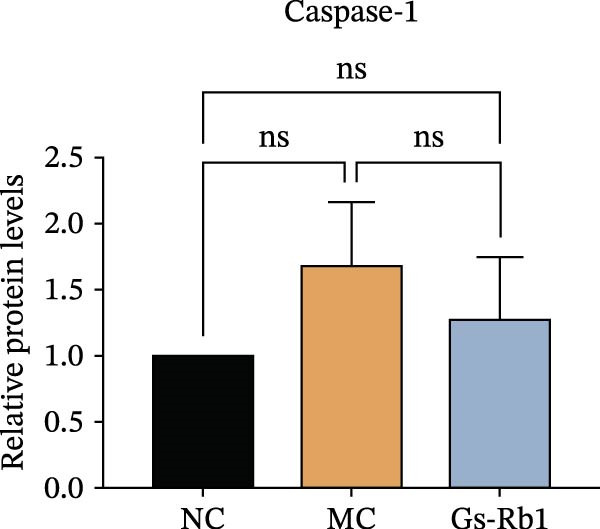
(b)

**Figure:**
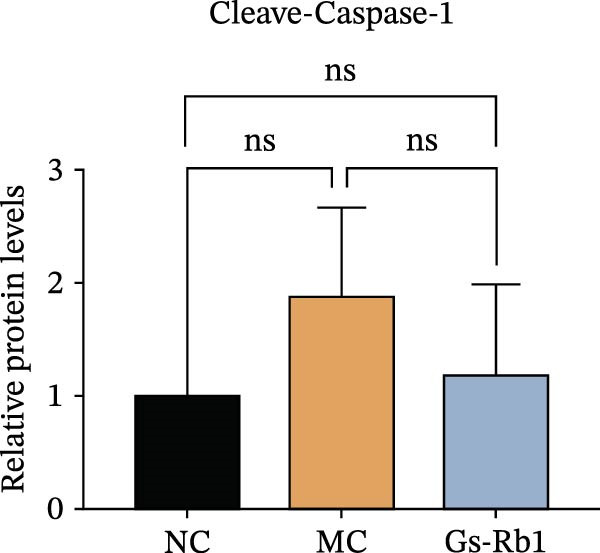
(c)

**Figure:**
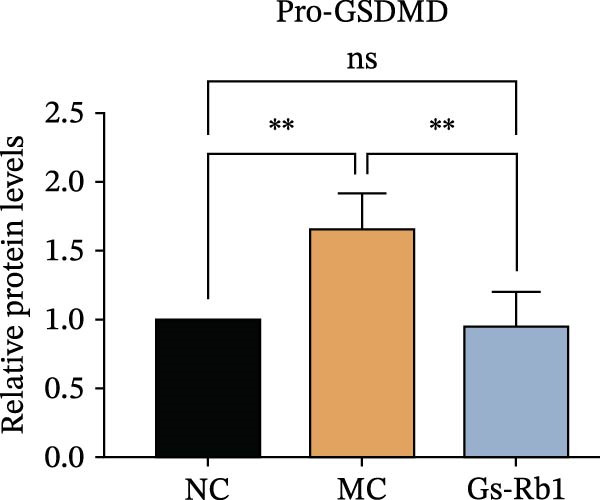
(d)

**Figure:**
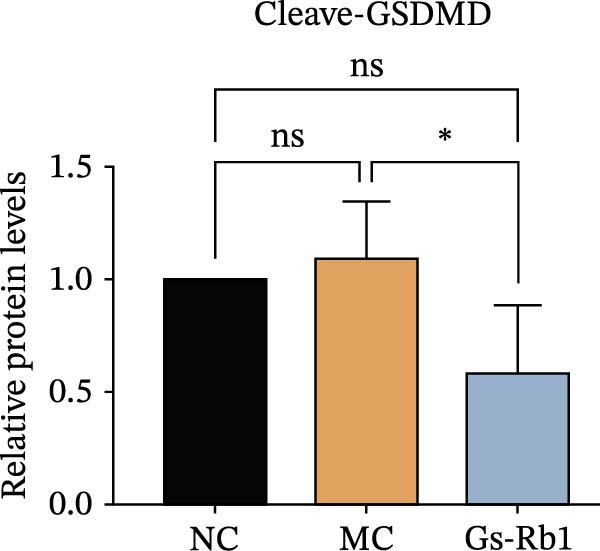
(e)

**Figure:**
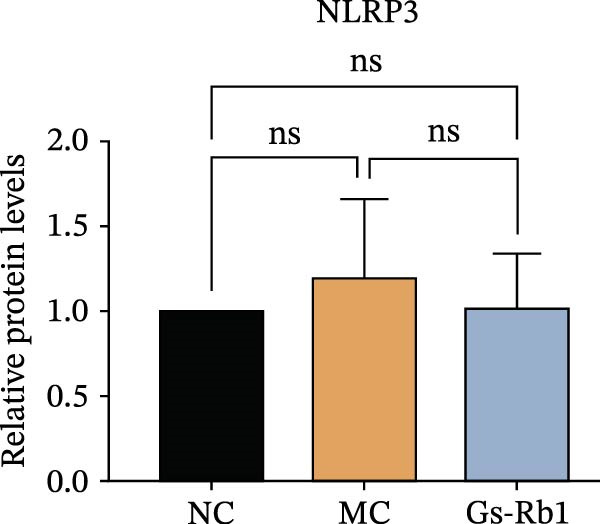
(f)

**Figure:**
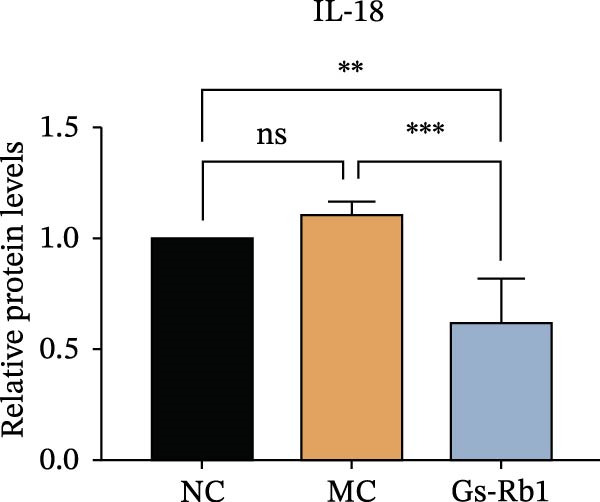
(g)

**Figure:**
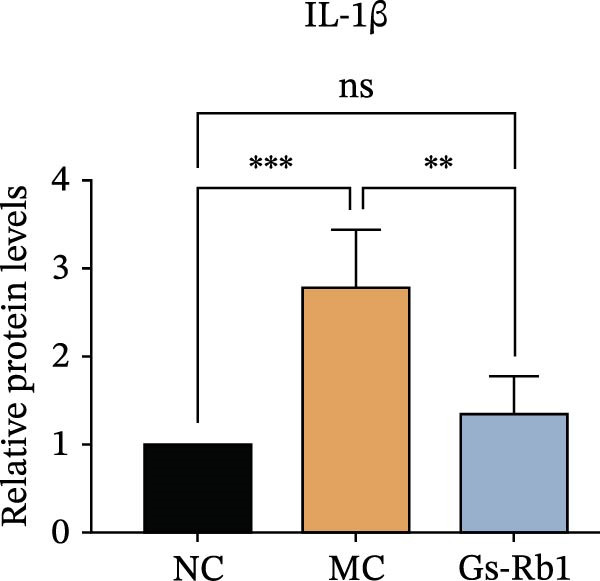
(h)

**Figure:**
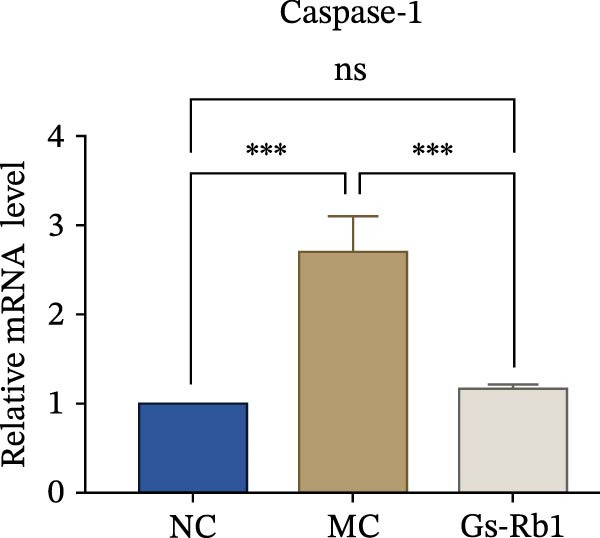
(i)

**Figure:**
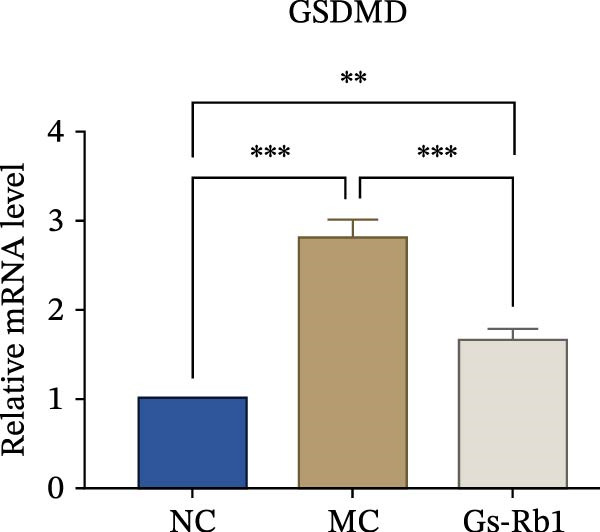
(j)

**Figure:**
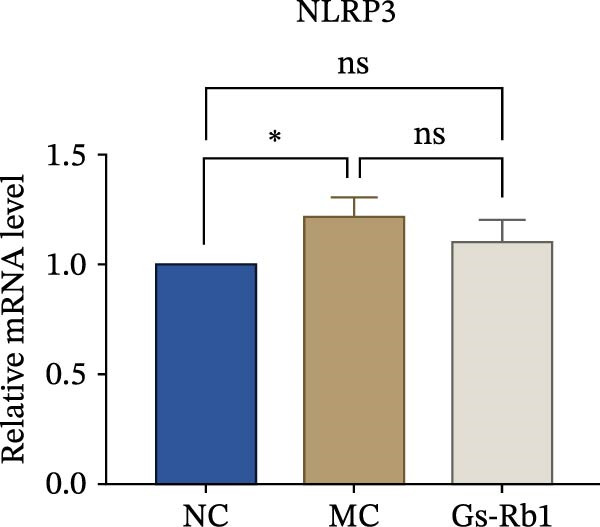
(k)

**Figure:**
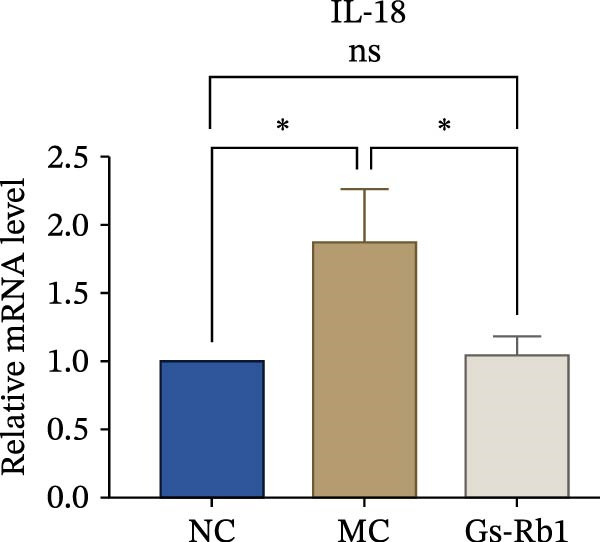
(l)

**Figure:**
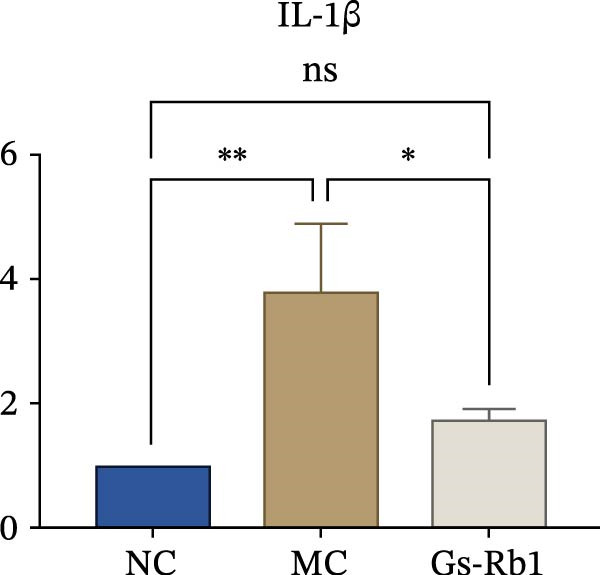
(m)

**Figure:**
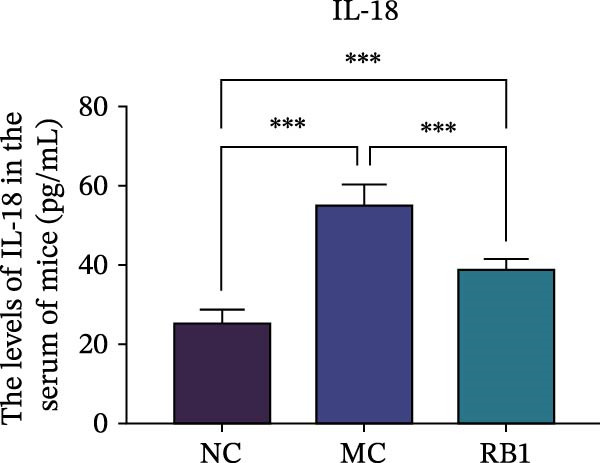
(n)

**Figure:**
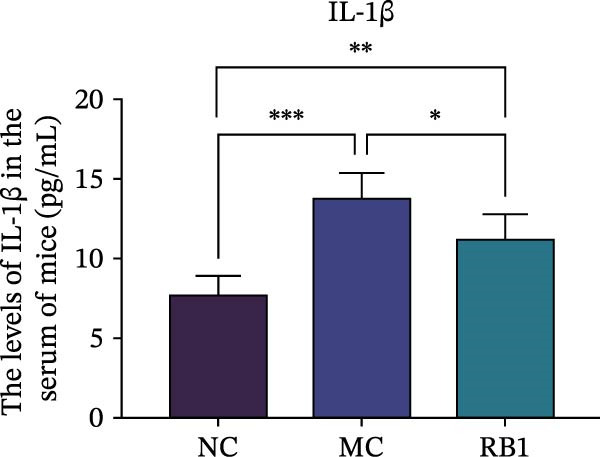
(o)

**Figure:**
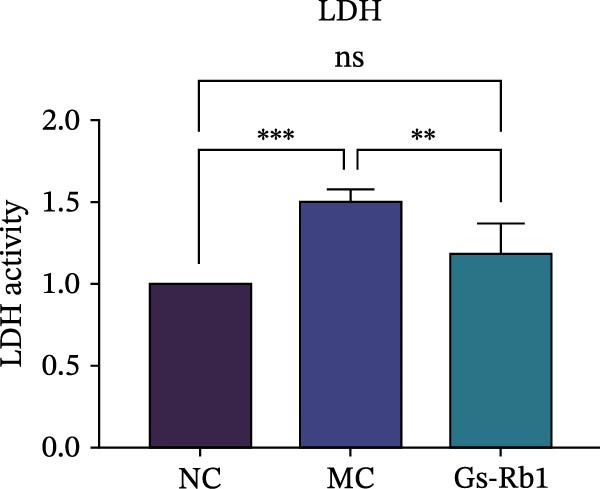
(p)

In addition, we also detected the levels of inflammatory factors and lactate LDH in ApoE^−/−^ mice serum by ELISA. The experimental results show that Gs‐Rb1 could reduce the levels of IL‐18, IL‐1β, and LDH in the serum of ApoE^−/−^ mice (Figure 3n–p). Following Gs‐Rb1 treatment, LDH expression was reduced to 78.6% of the level observed in the MC; IL‐18 was 70.5%; and IL‐1β was 81.9%. It is further proved that Gs‐Rb1 has a certain effect on reducing the level of pyroptosis in ApoE^−/−^ mice.

Although the current study demonstrated significant anti‐atherosclerotic efficacy of Gs‐Rb1 at 40 mg/kg/day, a formal dose–response curve was not established in vivo. Preliminary data from a short‐term pilot study (2 weeks, *n* = 3 per dose) suggested that 40 mg/kg/day is the minimal effective dose, but systematic evaluation of lower and higher doses (e.g., 10–80 mg/kg/day) over the full 12‐week protocol will be undertaken in future work to define the optimal therapeutic window.

### 3.5. Ginsenoside Rb1 Reduces Cell Membrane Rupture and Endothelial Cell Pyroptosis

To further confirm that Gs‐Rb1 has a reducing effect on EC pyroptosis, MAECs were used for the study. The experimental results show that ox‐LDL can induce pyroptosis of EC; cell membrane rupture was obvious in the MC group. After the Gs‐Rb1 intervention, the pyroptotic rate of cells decreased, and the effect of Gs‐Rb1−80 μg/mL was the most obvious (Figure 4a–f). While Annexin V/PI staining indicates membrane phosphatidylserine exposure and permeabilization, it cannot unequivocally discriminate pyroptosis from late apoptosis; therefore, cleaved GSDMD and active caspase‐1 immunoblots were additionally performed (Figure 5) to corroborate pyroptotic cell death.

Figure 4Gs‐Rb1 can improve cell death levels and reduce cell death rate. (a–e) BLANK, NC, MC, Gs‐Rb1‐60 μg/mL, and Gs‐Rb1−80 μg/mL, respectively. NC (normal control), MC (ox‐LDL model control), Gs‐Rb1L (low‐dose Rb1, 60 μg/mL), and Gs‐Rb1H (high‐dose Rb1, 80 μg/mL). (f) Percentage of pyroptosis. ^∗^ *p* < 0.05,  ^∗∗^ *p* < 0.01, NS, non‐significant, *n* = 3 biologically independent animals per group for all datasets shown in this panel (NC, MC, Gs‐Rb1L, and Gs‐Rb1H).

**Figure:**
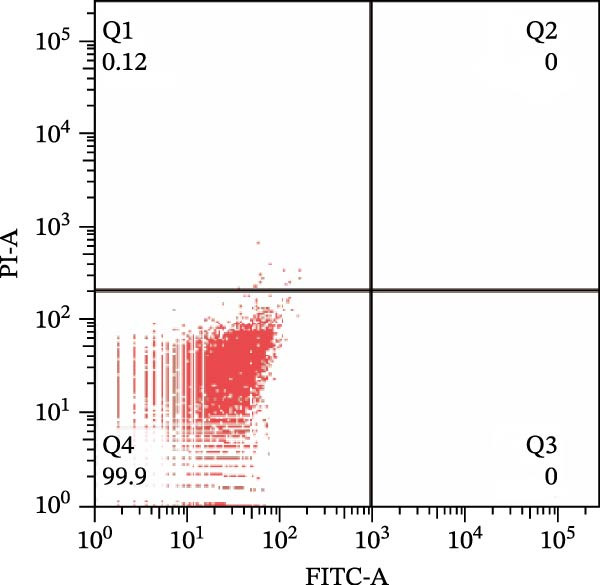
(a)

**Figure:**
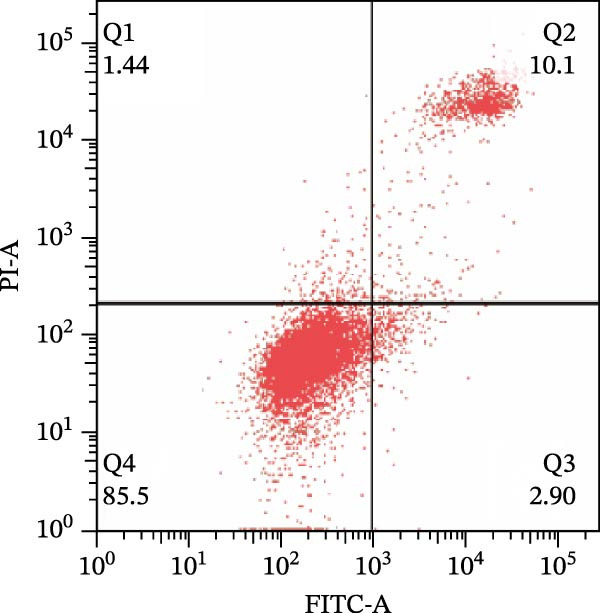
(b)

**Figure:**
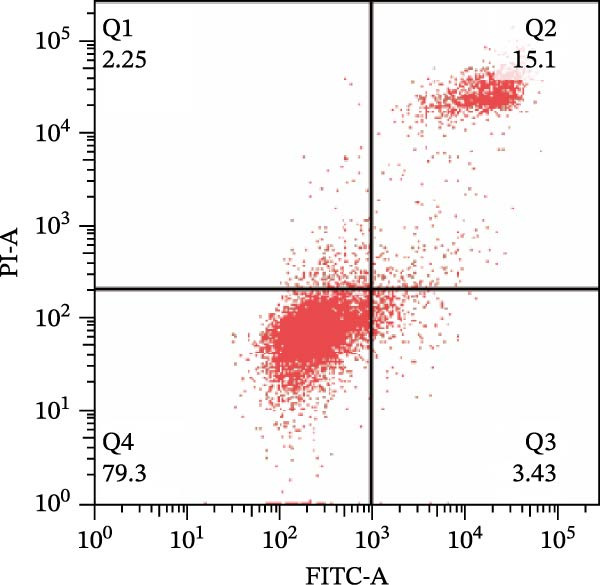
(c)

**Figure:**
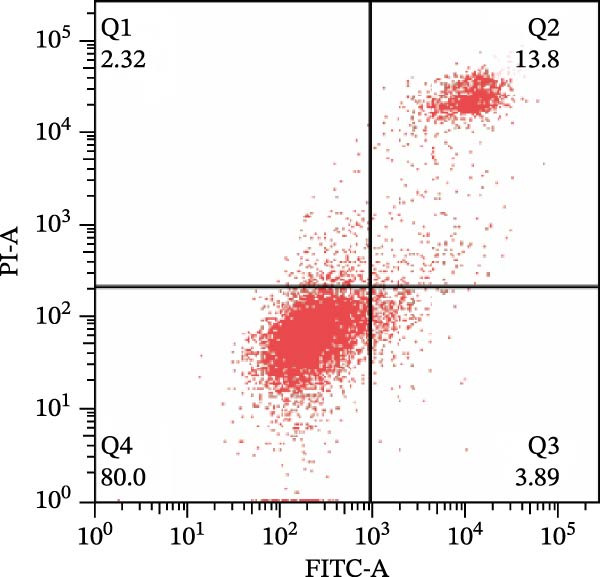
(d)

**Figure:**
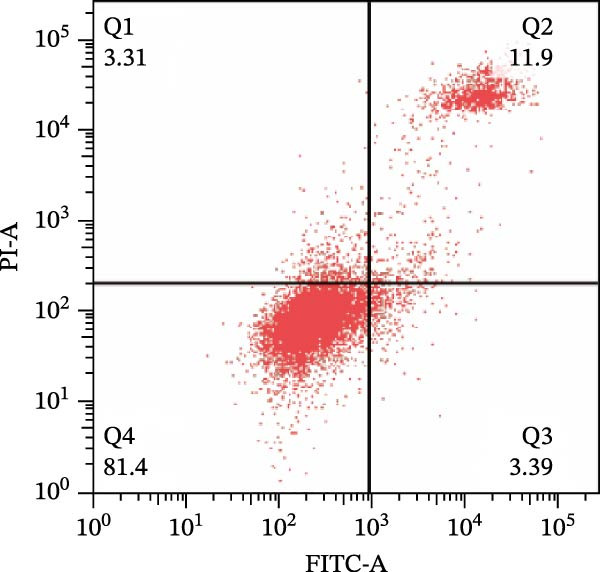
(e)

**Figure:**
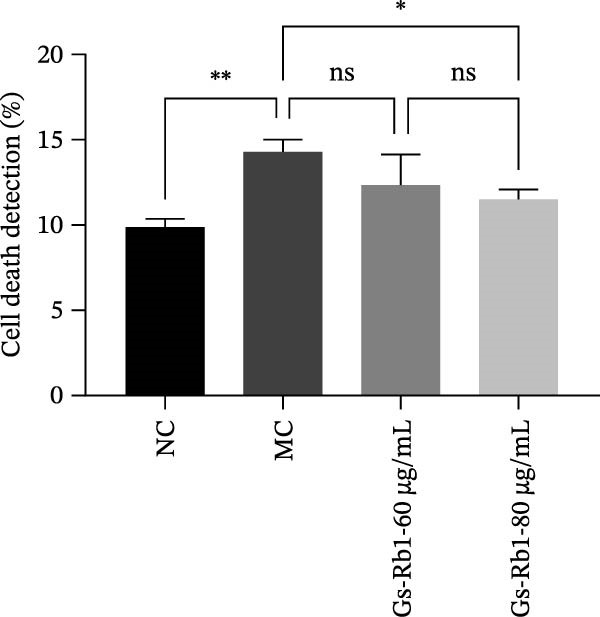
(f)

Figure 5Gs‐Rb1 reduce the level of pyroptosis in MAEC. (a–f) The protein levels of pyroptosis in MAEC. (g–l) Gene expression levels of pyroptosis in MAEC.  ^∗^ *p* < 0.05,  ^∗∗^ *p* < 0.01,  ^∗∗∗^ *p* < 0.001, NS, non‐significant, *n* = 4 biologically independent animals per group for all datasets shown in this panel (NC, MC, Gs‐Rb1L, and Gs‐Rb1H).

**Figure:**
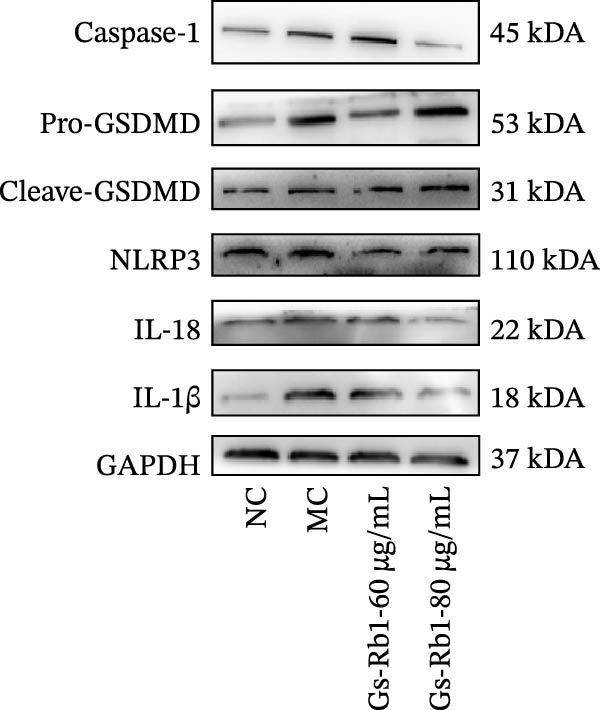
(a)

**Figure:**
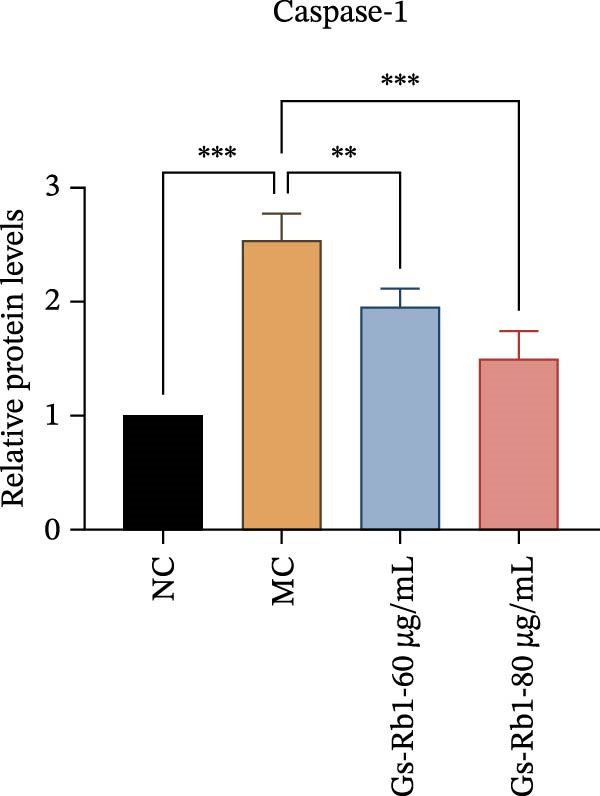
(b)

**Figure:**
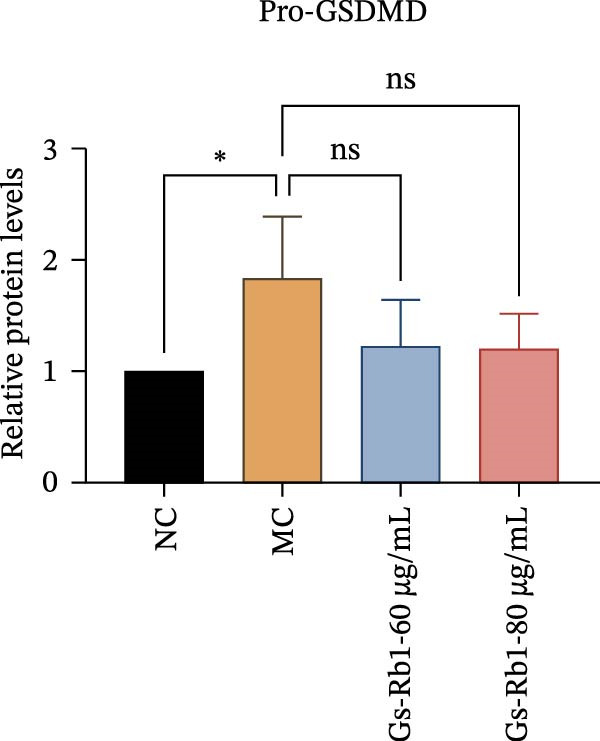
(c)

**Figure:**
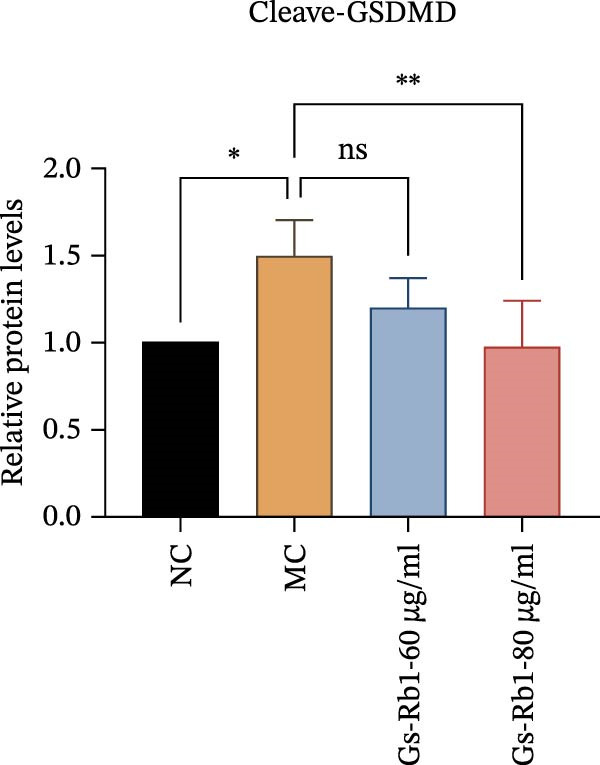
(d)

**Figure:**
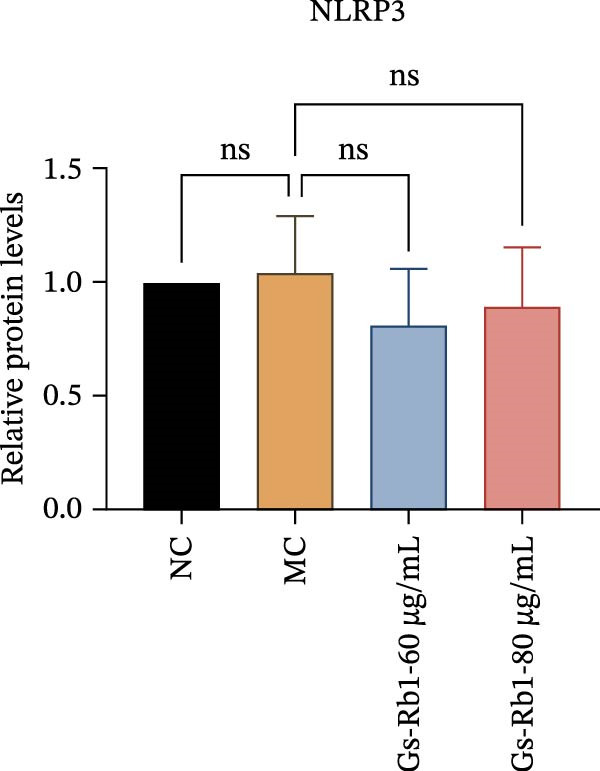
(e)

**Figure:**
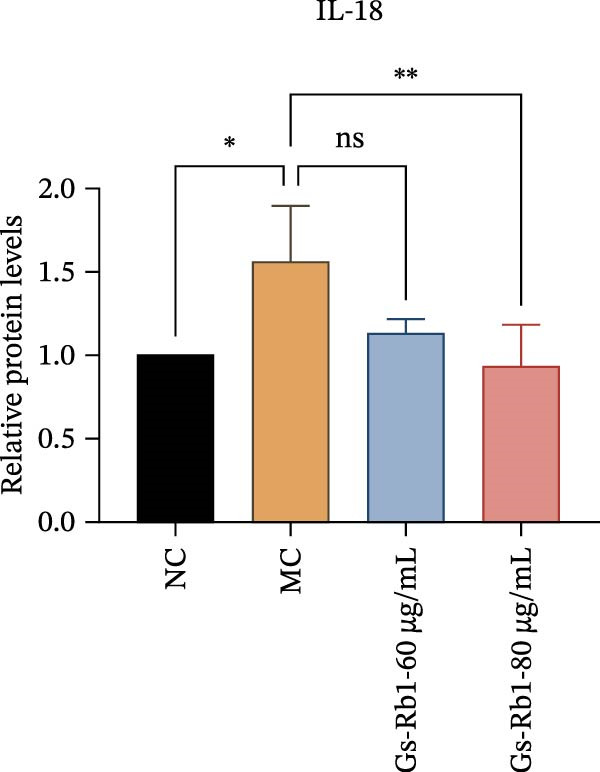
(f)

**Figure:**
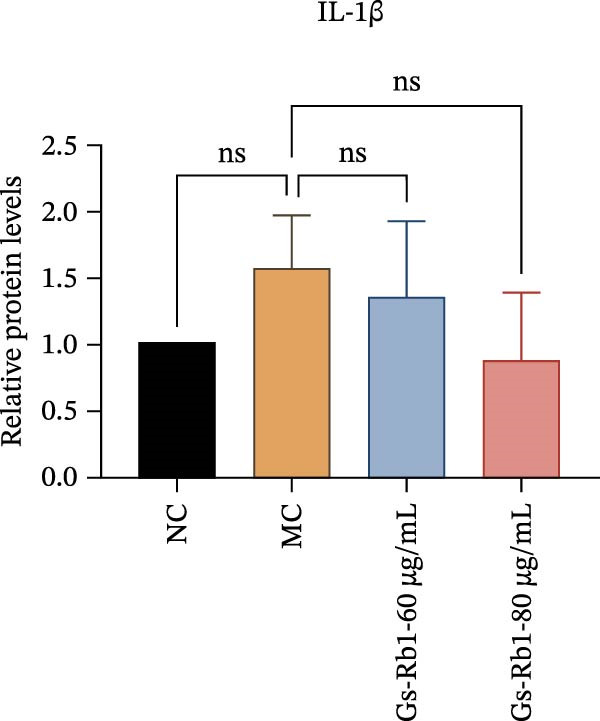
(g)

**Figure:**
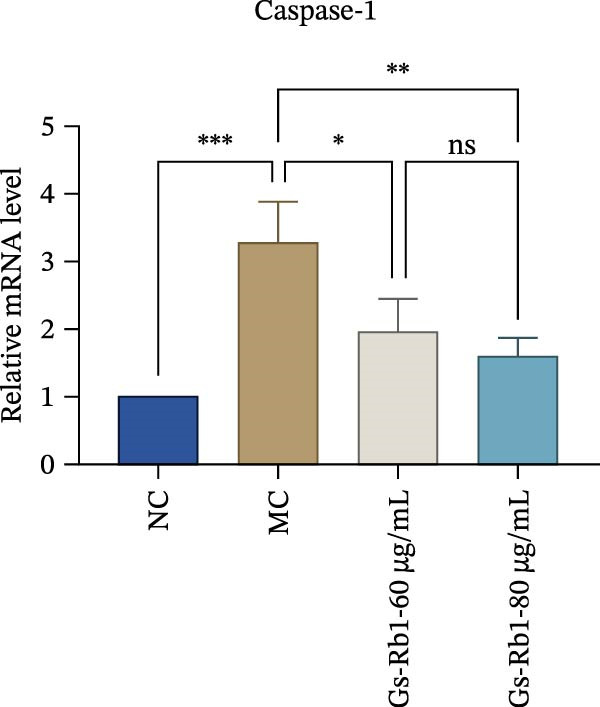
(h)

**Figure:**
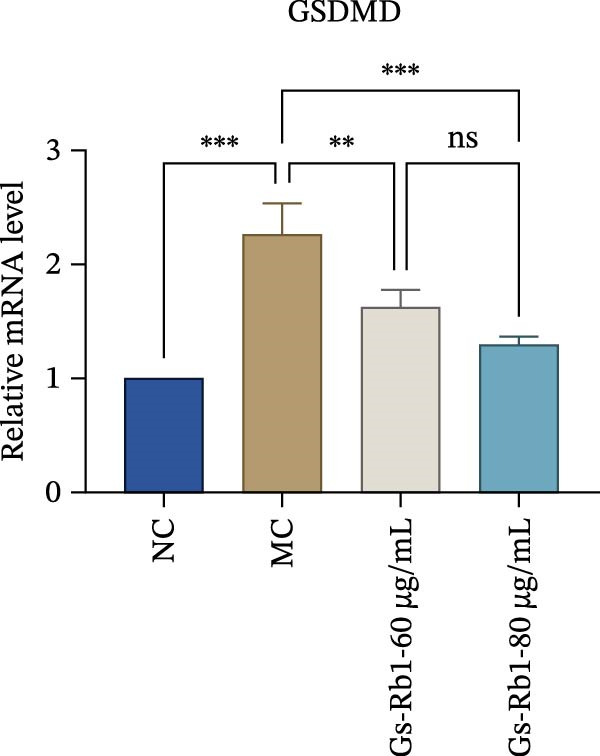
(i)

**Figure:**
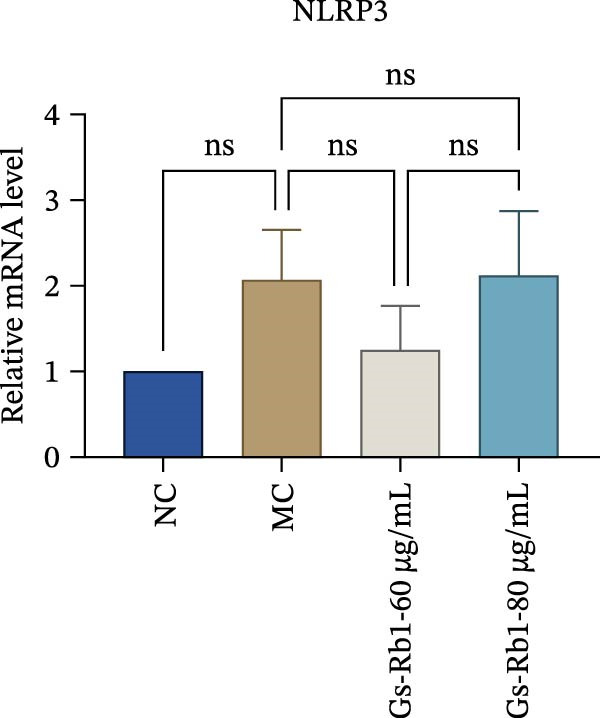
(j)

**Figure:**
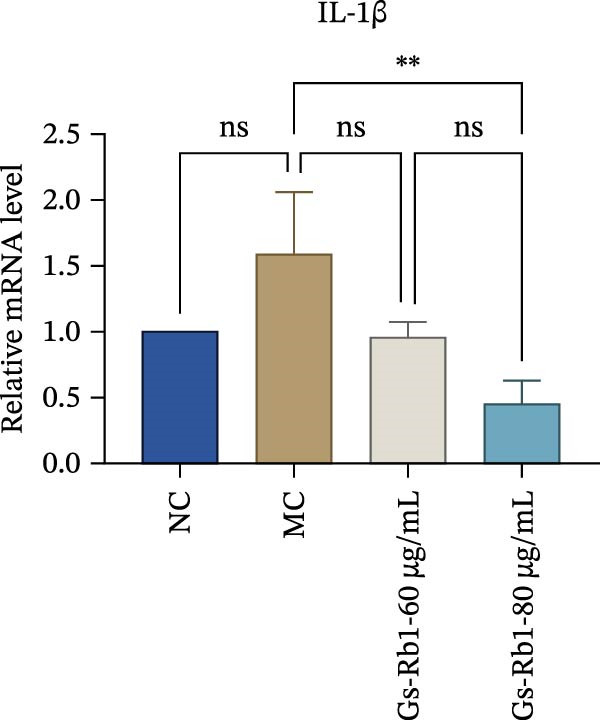
(k)

**Figure:**
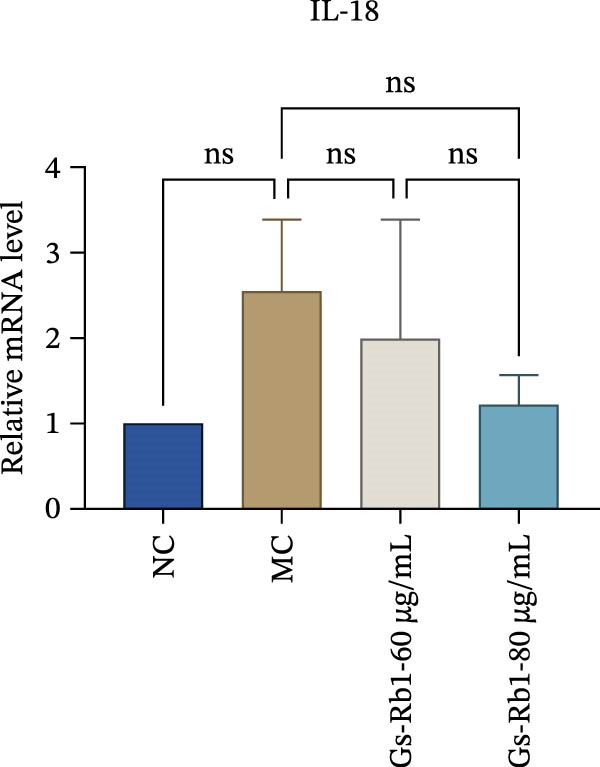
(l)

### 3.6. Ginsenoside Rb1 Reduced the Pyroptosis‐Related Protein in Endothelial Cells

To further verify the effect of Gs‐Rb1 on EC pyroptosis, this experiment used Western blot to detect changes in pyroptosis‐related indicators in EC. The classic pyroptosis pathway mediated by caspase‐1 plays a key role in the development of AS. The inflammasome binds to caspase‐1 and promotes its activation, thereby mediating GSDMD cleavage and promoting the formation of cell membrane pores [19]. The experimental results showed that compared with the NC group, the protein content of caspase‐1 in the MC group was significantly increased, indicating that after damage by ox‐LDL, the level of cell pyroptosis in the MC group increased significantly. After treatment with different doses of Gs‐Rb1, the protein content of caspase‐1 showed a downward trend (Figure 5b). Compared with MC, the protein expression of caspase‐1 was reduced by 77% and 59.1% in Gs‐Rb1L and Gs‐Rb1H, respectively. Despite this reduction, caspase‐1 levels in both Gs‐Rb1‐treated groups remained significantly higher than those in the NC group (*p* < 0.01), indicating that Gs‐Rb1 exerts a partial rather than complete inhibitory effect on caspase‐1 activation. This suggests that while Gs‐Rb1 can mitigate pyroptotic signaling, it may not fully restore caspase‐1 to physiological baseline levels under persistent hyperlipidemic stress. Whether this reflects a limitation in therapeutic efficacy or a dose‐dependent effect remains to be determined. The levels of GSDMD and Cleave‐GSDMD, which are key substrates for cell pyroptosis, were also reduced by Gs‐Rb1 (Figure 5c,d). Compared with the MC, Cleave‐GSDMD decreased by 80.4% and 65.32%, and pro‐GSDMD decreased by 67% and 65.7% in the Gs‐Rb1L and Gs‐Rb1H groups, respectively. NLRP3, as one of the most critical inflammasomes in the occurrence of pyroptosis, can promote the activation of caspase‐1 and inflammatory factors. Experimental results show that Gs‐Rb1 can decrease the expression of NLRP3 and the release of IL‐18 and IL‐1β is significantly reduced at the same time (Figure 5e,g). After ginsenoside treatment, NLRP3 levels decreased by 77.4% and 85.1%, respectively, compared with MC.

In addition, we detected the expression changes of pyroptosis‐related genes in MAECs through realtime PCR. The experimental results showed that compared with the NC group, the mRNA of caspase‐1 in the MC group was increased. After treatment with Gs‐Rb1, the mRNA of caspase‐1 was significantly decreased by 60% and 35.6% (Figure 5h). In addition, GSDMD also has an obvious downward trend (Figure 5i). Compared with the MC, levels decreased by 72% and 57.5%, respectively. However, there is no obvious downward trend in inflammasome NLRP3. Compared with the MC group, there was no significant change in the Gs‐Rb1 treatment group (Figure 5j). Besides, compared with the MC group, although the expression of IL‐18 decreased, but there was no significant statistical difference (Figure 5k). The expression of IL‐1β was significantly reduced with Gs‐Rb1 at 80 μg; compared with MC, it decreased by 28.6% (Figure 5l).

## 4. Discussion

AS is an inflammatory reaction caused by lipid metabolism disorder, which can cause various cardiovascular diseases such as coronary heart disease and stroke [20]. Endothelial dysfunction caused by hemodynamic disturbances [21] and hypercholesterolemia [20] is the initial stage of AS. Cell death and inflammation contribute significantly to the development of AS. Pyroptosis is a new type of cell death characterized by the cell membrane being broken and inflammatory cytokines are released [22]. GSDMD can be specifically cleaved by inflammatory caspases (such as caspase‐1 and caspase‐11) after activation of inflammasomes. This cleavage process is a key step in the occurrence of cell pyroptosis. After cutting, Cleave‐GSDMD is released and transferred to the cell membrane to form a poly pore structure, which leads to an increase in the permeability of the cell membrane, the release of cell contents, and ultimately triggers pyroptosis of the cell. This process is critical for clearing intracellular pathogens and activating the inflammatory response. In recent years, studies have found that pyroptosis plays an important role in the occurrence and development of infectious diseases [22], metabolic diseases [23], AS [24], and other diseases. ECs constitute a metabolically active interface between blood and the vessel wall; their pyroptosis facilitates lipid deposition and thrombus formation on the intima. Consequently, preventing or treating vascular EC pyroptosis has emerged as a key research area.

*Panax ginseng* C.A. Meyer is grown in China, Japan, North Korea, South Korea, and Russia [25]. Roots are mainly used for medicinal purposes. As a representative of traditional Chinese medicine for more than 5000 years [9]. Ginseng has various therapeutic effects, such as invigorating Qi, nourishing blood, strengthening the spleen, and benefiting the lungs. Ginseng contains various active substances such as saponins, peptides, volatile oils, and polysaccharides. Among them, saponins are considered to be the main components responsible for the pharmacological activity [26]. Gs‐Rb1 is a dammarane‐type triterpene saponin compound with significant cardioprotective effects. For example, Gs‐Rb1 can play a cardioprotective role by being anti‐oxidative, anti‐inflammatory, and antiapoptotic, promoting angiogenesis and improving circulation in myocardial ischemia‐reperfusion [27]. In addition, Gs‐Rb1 exerts antiarrhythmic effects by relieving calcium overload and protecting cardiomyocytes [13].

Gs‐Rb1 has been shown to have a significant anti‐atherosclerotic effect in previous studies. Gs‐Rb1 promotes the transformation of LC3 from type I to type II, thereby accelerating autophagy to inhibit apoptosis. These findings suggest that Gs‐Rb1 plays a therapeutic role in AS by regulating the balance between apoptosis and autophagy [28]. In addition, Gs‐Rb1 enhanced the stability of atherosclerotic plaques by reducing lipid accumulation and inducing autophagy in macrophages [26]. The above studies demonstrate that Gs‐Rb1 has great potential in the treatment of AS.

Although previous studies have reported the anti‐atherosclerotic effects of Gs‐Rb1, most have focused on its roles in apoptosis inhibition, autophagy regulation, or lipid metabolism modulation in macrophages or smooth muscle cells. For instance, Zhou et al. [28] demonstrated that Gs‐Rb1 inhibits early AS by enhancing autophagy and reducing apoptosis in ApoE^−/−^ mice. Similarly, Qiao et al. [29] showed that Gs‐Rb1 stabilizes atherosclerotic plaques by promoting lipid efflux and autophagy in macrophages.

However, few studies have specifically investigated the role of Gs‐Rb1 in endothelial pyroptosis, a newly recognized form of inflammatory cell death that contributes to endothelial dysfunction and plaque initiation. A recent study highlighted the therapeutic potential of targeting pyroptosis in AS, while others have emphasized the importance of NLRP3 inflammasome and caspase‐1 pathways in vascular inflammation. Apart from ECs, atherosclerotic plaques are populated by macrophages, smooth‐muscle cells, and lymphocytes. Given the broad pharmacological spectrum of Gs‐Rb1, it is plausible that the compound also modulates the inflammatory phenotype of these non‐target cells. For instance, Zhou et al. [17] reported that Rb1 enhances autophagy and reduces lipid accumulation in macrophage foam cells, indirectly stabilizing plaques. Conversely, off‐target modulation of osteoclast/osteoblast signaling might parallel the well‐documented skeletal effects of statins. A recent meta‐analysis [30] revealed that statin therapy produces a modest but significant increase in bone mineral density in patients with cardiovascular disease, presumably via inhibition of the mevalonate pathway and downstream inflammatory cytokines. Should future studies confirm similar systemic activity for Gs‐Rb1, its therapeutic index in AS may need to be re‐evaluated in light of both vascular and extra‐vascular endpoints, including bone‐metabolic markers.

**Figure 6:**
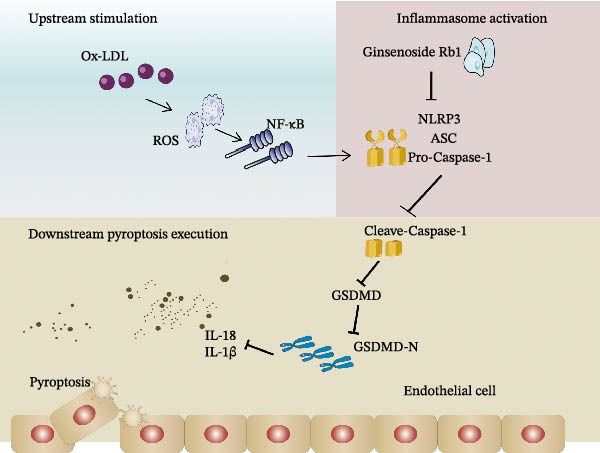
Mechanism of action of Gs‐Rb1 on endothelial cell pyroptosis.

Therefore, our study fills a critical research gap by providing the first evidence that Gs‐Rb1 attenuates AS by inhibiting endothelial pyroptosis, particularly through the NLRP3/caspase‐1/GSDMD axis. This mechanistic insight not only expands the understanding of Gs‐Rb1s cardiovascular protective effects but also supports the development of endothelial‐targeted anti‐pyroptotic therapies for AS. To better integrate our findings into a mechanistic framework, we propose a working model (Figure 6) illustrating the potential sequence of action: Gs‐Rb1 may suppress upstream priming signals (e.g., ROS or NF‐κB activation), thereby inhibiting NLRP3 inflammasome assembly and caspase‐1 activation. This leads to reduced GSDMD cleavage, diminished membrane pore formation, and ultimately decreased secretion of IL‐1β and IL‐18. While direct binding or molecular interaction remains to be validated, this model provides a testable hypothesis for future studies investigating the precise mode of action of Gs‐Rb1 in endothelial pyroptosis inhibition.

In this study, we provide systematic evidence that Gs‐Rb1 significantly inhibits NLRP3‐mediated endothelial pyroptosis and attenuates AS progression. In ApoE^−/−^ mice, 12‐week treatment with Gs‐Rb1 (40 mg/kg/day) reduced aortic root plaque area by 42.3% compared to the model group (*p* < 0.01, Figure 1). Immunofluorescence and Western blot analyses showed that Gs‐Rb1 decreased cleaved caspase‐1 expression by 61.8% (*p* < 0.001), GSDMD‐N by 53.4% (*p* < 0.01), and NLRP3 protein by 14.3% (*p* < 0.05, Figure 3). Serum levels of inflammatory cytokines were also significantly reduced: IL‐1β by 18.1% (*p* < 0.01), IL‐18 by 29.5% (*p* < 0.01), and LDH release by 21.4% (*p* < 0.05). In ox‐LDL–stimulated MAECs, treatment with 80 μg/mL Gs‐Rb1 for 12 h reduced the cell death rate from 38.7% to 19.4% (*p* < 0.001, Figure 4), cleaved GSDMD expression by 65.3% (*p* < 0.001), and caspase‐1 activity by 59.1% (*p* < 0.01, Figure 5). These quantitative findings consistently demonstrate that Gs‐Rb1 alleviates endothelial pyroptosis via the NLRP3/caspase‐1/GSDMD axis, representing a key mechanism underlying its anti‐atherosclerotic effect.

Collectively, our findings not only confirm the anti‐atherosclerotic efficacy of Gs‐Rb1 but also, for the first time, elucidate its mechanistic role in inhibiting NLRP3‐mediated endothelial pyroptosis. These results provide novel experimental support for Gs‐Rb1 as a potential therapeutic agent in AS and highlight the targeting of pyroptosis as a promising strategy for vascular protection.

Finally, our observed reductions in IL‐1β and IL‐18 fit well within the broader anti‐inflammatory profile of ginseng. A 2024 systematic review and meta‐analysis of randomized controlled trials [30] concluded that ginseng supplementation significantly lowers hs‐CRP and IL‐6 in adults with cardiovascular risk factors. By extending the evidence to the NLRP3/caspase‐1/IL‐1β axis, the present study provides mechanistic insight that may guide future dose‐optimization studies and strengthen the biological plausibility of ginseng‐based cardioprotective interventions.

However, our research still has certain limitations. First, the study design lacks a critical control group—ApoE^−/−^ mice fed with a standard diet (chow diet). In the current experimental design, we compared C57BL/6J mice (normal control, NC) with ApoE^−/−^ mice fed HFD, with the latter being randomly assigned to model control (MC) and Gs‐Rb1 treatment groups; thus, without an ApoE^−/−^ + standard diet group, we cannot definitively distinguish whether the observed atherosclerotic phenotypes and pyroptosis markers are primarily driven by the ApoE genetic deficiency itself, the high‐fat dietary challenge, or their synergistic interaction, which precludes us from isolating the specific contribution of HFD‐induced metabolic stress versus basal pathological changes conferred by the ApoE knockout. Future studies should incorporate this essential control arm to better delineate the independent and combined effects of genetic susceptibility and dietary intervention on endothelial pyroptosis and plaque formation. Second, no drug dose analysis was performed in animal experiments, and future studies should optimize the drug dosing regimen in subsequent studies. Third, although reduced caspase‐1 cleavage and IL‐1β release strongly suggest that Gs‐Rb1 interferes with the NLRP3 inflammasome, the absence of a selective inhibitor (e.g., MCC950) or gene‐knockdown strategy limits causal inference. Follow‐up studies incorporating MCC950 or NLRP3^−/−^ cells will be required to confirm that Rb1‐mediated protection is indeed NLRP3‐dependent. Fourth, key experiments (e.g., flow‐cytometry cell death assays and Western blots) were performed with 3–4 biological replicates per group, which may be underpowered and increase the risk of type II error. Future studies should perform a priori sample‐size calculations and use ≥6–8 replicates to enhance robustness. Fifth, current outcomes focused on plaque area, protein expression, and serum cytokines; clinically relevant indices such as plaque rupture, thrombosis, or survival were not assessed. Finally, pharmacokinetic profiling in larger animals and humans is lacking. Future studies should determine plasma half‐life, tissue distribution, and metabolite activity to guide dose selection and dosing frequency. It is hoped that more detailed experimental grouping can be carried out in subsequent studies.

## 5. Conclusion

In summary, Gs‐Rb1 retards AS progression in ApoE^−/−^ mice by inhibiting endothelial pyroptosis through the NLRP3/caspase‐1/GSDMD axis. Given its oral efficacy at 40 mg/kg/day, favorable safety profile in rodent and primate studies, and readily scalable purification, Gs‐Rb1 is a credible candidate for early‐phase human trials. A randomized, placebo‐controlled translational study evaluating endothelial function and systemic inflammatory markers in patients with sub‐clinical AS is now being planned to bridge the current pre‐clinical findings to clinical practice.

## Author Contributions

Conceptualization: Xuejiao Jiang and Wenjing Zong. Writing – original draft: Xiongyao Wang. Formal analysis: Chun Chen and Xinyu Ji. Investigation and validation: Tianqi Jiang and Zhaoyu Pan.

## Funding

This study was supported by the Inner Mongolia Medical University Science and Technology Project (YKD2020KJBW017 and YKD2024QN003); the Natural Science Foundation of Inner Mongolia Autonomous Region (2024QN08048); and the Beijing Natural Science Foundation (7254535).

## Conflicts of Interest

The authors declare no conflicts of interest.

## Supporting Information

Additional supporting information can be found online in the Supporting Information section.

## Supporting information


**Supporting Information 1** Table S1: List of forward and reverse primer sequences for all genes analyzed by RT‐qPCR in this study.

**Supporting Information 2** Figure S1A: Body weight changes in ApoE^−/−^ mice during the experimental period. (a) Body weight was measured weekly in normal control (NC), model control (MC), and ginsenoside Rb1 (Gs‐Rb1) treatment groups. Data are presented as mean ± SEM (*n* = 5 per group).

## Data Availability

The data that support the findings of this study are available from the corresponding author upon reasonable request.
