# Supplementary figures and images for: Ginsenoside Rb1 Improves Atherosclerosis by Inhibiting Endothelial Cell Pyroptosis
Source: Oxid Med Cell Longev. 2026 Apr 29;2026:6137635. doi: 10.1155/omcl/6137635 (PMC13129223; doi:10.1155/omcl/6137635)

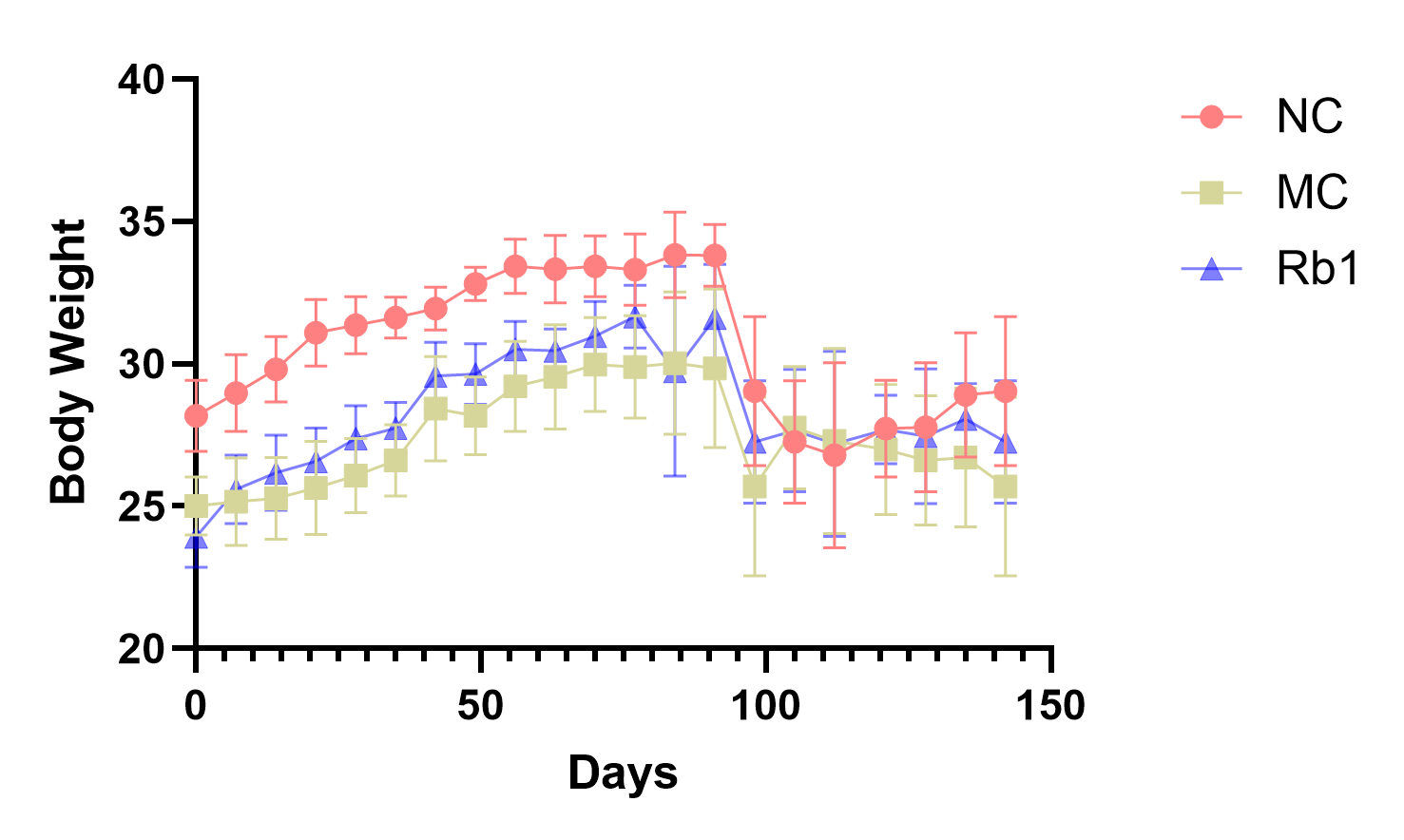

Supplement: Supplementary file 2 — Supporting Information 2 Figure S1A: Body weight changes in ApoE−/− mice during the experimental period. (a) Body weight was measured weekly in normal control (NC), model control (MC), and ginsenoside Rb1 (Gs‐Rb1) treatment groups. Data are presented as mean ± SEM (n = 5 per group). [file OMCL-2026-6137635-s001.tif]
